# Syndrome of inappropriate anti-diuretic hormone in non-small cell lung carcinoma: a case report

**DOI:** 10.3332/ecancer.2012.279

**Published:** 2012-11-14

**Authors:** Philip McDonald, Colleen Lane, Graciela E Rojas, Ashiq Masood

**Affiliations:** 1 School of Medicine, Wayne State University, Detroit, MI 48201, USA; 2 Detroit Medical Center, Wayne State University, Detroit, MI 48201, USA; 3 Division of Hematology/Oncology, Department of Medicine, University of Maryland Greenabaum Cancer Center, 22 S. Greene St., Room N9E17, Baltimore, MD 21201, USA

## Abstract

Paraneoplastic syndrome (PNS) related to lung cancer is very common. However, the syndrome of inappropriate anti-diuretic hormone secretion (SIADH) is rare in non-small cell lung cancer (NSCLC). We are reporting the case of a 58-year-old female presenting with dyspnea, cough, weight loss, digital clubbing, and one week of haemoptysis. CT showed a mediastinal mass completely encasing her superior vena cava, causing significant narrowing of the trachea and right mainstem bronchus. Bronchoscopy and biopsy identified a non-resectable NSCLC. Palliative radiation therapy was initiated. The day after her first radiation treatment, the patient developed asymptomatic hyponatremia, confirmed to be SIADH by laboratory evaluation. NSCLC-associated SIADH has been reported only thrice over the past two decades and never following radiation therapy with clinical improvement. The patient was discharged home on fluid restriction after her respiratory status improved to continue outpatient radiation and chemotherapy. SIADH in the setting of NSCLC is discussed.

## Introduction

The syndrome of inappropriate anti-diuretic hormone secretion (SIADH) is characterized by hyponatremia caused by retention of free water secondary to dysregulated release of anti-diuretic hormone (ADH) [1].

Syndrome of inappropriate anti-diuretic hormone secretion (SIADH) was first associated with malignancy when described in two patients with bronchogenic carcinoma in 1957 [2]. We now know that it is a common paraneoplastic phenomenon and that approximately 70% of malignancy-related cases are the result of small cell lung cancer (SCLC) [3]. Lymphoma, thymoma, mesothelioma, Ewing’s sarcoma, and a variety of carcinomas including squamous cell carcinoma of the head and neck have all been associated with the development of SIADH [4].

Non-small cell lung cancer (NSCLC) is a very rare cause of SIADH, with only three published cases over the past two decades [5–7]. In one large case series, only 0.7% of 427 patients with NSCLC were diagnosed with the syndrome [8]. In this report, we present a case of SIADH which developed in a patient with NSCLC following initiation of palliative radiation therapy. This case is unique in that hyponatremia worsened concomitantly with clinical improvement and decreased tumour burden, suggesting release of ADH from malignant cells during tumour lysis, a finding only previously described in SCLC [3].

## Case report

A 58-year-old woman presented with a one-month history of a cough and fatigue, and a one-week history of haemoptysis. She reported a 20 Ib weight loss over the last year. She had a 45 pack-per-year smoking history. Her past medical history was significant for untreated hepatitis C and alcoholism. Family history included a father and sister with an unknown type of cancer.

At presentation, the patient was afebrile, normotensive, and breathing comfortably at 16 breaths per min while at rest. A complete blood count showed a haemoglobin of 12.0 gm/dl (normal 12.1–15.1). Her white blood cell count, platelets, and hematocrit were all within normal limits. Her electrolyte panel showed mild hyponatremia (serum sodium level of 132 mmol/l). The rest of her electrolytes were unremarkable. The patient’s physical exam was significant for hypertrophic osteoarthropathy in all of her fingers. Her pulmonary exam revealed decreased breath sounds over the right lung fields compared to the left lung fields. There was also diffuse wheezing over the right posterior lung fields.

**Figure 1:**
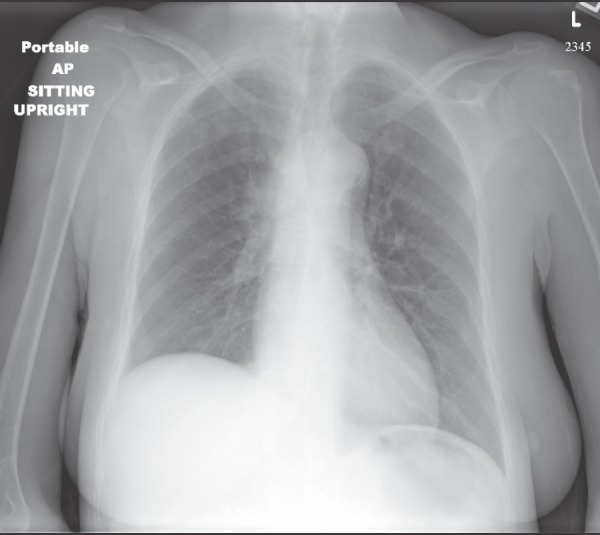
Chest x-ray demonstrating an elevated right hemi-diaphragm, mediastinal lymphadenopathy, and tracheal compression near the level of the carina.

**Figure 2a and b:**
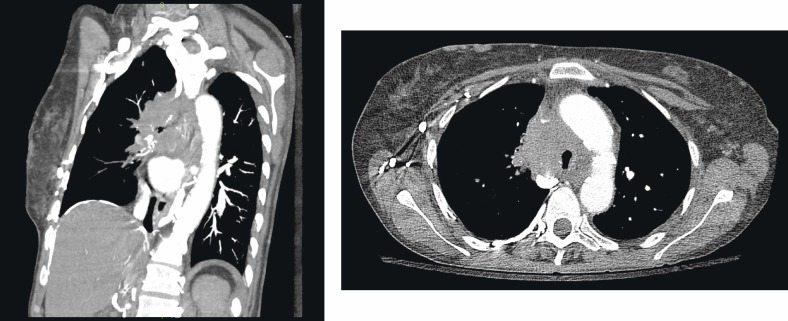
a) CT thorax demonstrating tracheal compression at the level of the tumor b) Lateral view showing the large mediastinal NSCLC with compression of the right mainstem bronchus.

A chest radiograph showed an elevated right hemi-diaphragm, mediastinal lymphadenopathy, as well as tracheal compression near the level of the carina (Figure 1). CT scan of chest showed a mediastinal mass completely encasing the patient’s superior vena cava (Figure 2a and b). The mass was causing significant narrowing of her trachea and right mainstem bronchus. The scan also revealed three right upper lobe lesions and a 2.6-cm mass in her left adrenal gland concerning for metastasis. MRI of the brain performed for staging showed no evidence of metastatic disease.

**Figure 3:**
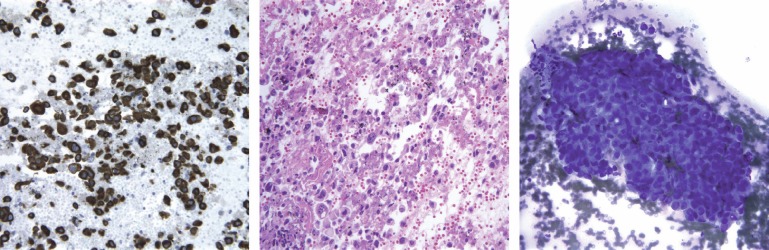
CK7 (cytokeratin 7), H&E, and Diff Quick staining of pathologic specimen. Morphology, positive CK7, and negative CD20 favoured adenocarcinoma of the lung.

The patient underwent bronchoscopy and biopsy as well as a fine needle aspiration of paratracheal lymph nodes. The pathology report showed tumour cells positive for CK7, negative for CK 5/6, p63, TTF-1, and CK20 immunostains. Lymphoid tissue in the lymph nodes was completely replaced by tumour. The combined morphologic features and immunoprofile were consistent with non-small cell carcinoma, favouring adenocarcinoma (Figure 3).

Due to the tracheal and right mainstem bronchus compression, there was concern for impending airway obstruction in this patient. Accordingly, emergency palliative radiation therapy to the mediastinum was instituted. After one treatment, her serum sodium level was noted to be 123 mmol/l, with a serum osmolality 262 mOsm/kg and a urine osmolality of 579 mOsm/kg. Clinically the patient was euvolemic, hemodynamically stable and asymptomatic. Based on these findings, the diagnosis of SIADH was made.

The patient in this case was treated with a fluid restriction of 1,000 ml/day. This strategy was successful in raising her serum sodium level to 126 mmol/l over the first 24 h. She continued to display no clinical symptoms of hyponatremia. Her serum sodium levels remained stable at 126 mmol over the remainder of her hospital stay.

After completing one week of radiation treatment, the patient began to experience improvement in her shortness of breath. On physical exam, there was improved air entry into her right lung. The patient was discharged home to complete the remaining five weeks of radiation treatment as an outpatient. Arrangements were also made for the patient to follow up with medical oncology to begin outpatient. Plans were made for further imaging, including a positron emission tomography (PET) scan to investigate of evidence of distant metastasis.

## Discussion

Syndrome of inappropriate anti-diuretic hormone secretion (SIADH) is characterized by hyponatremia caused by retention of free water secondary to dysregulated release of ADH [1]. While there are a number of causes of SIADH—pulmonary disorders including pneumothorax and status asthmaticus, CNS disturbances including stroke and haemorrhage, drugs such as tricyclic antidepressants (TCAs) and chemotherapeutic agents, and gain of function mutations in the V2 receptor—the majority of cases are caused by malignancy [4, 9]. SIADH was first associated with malignancy when described in two patients with bronchogenic carcinoma in 1957 [2]. We now know that it is a common paraneoplastic phenomenon and that approximately 70% of malignancy-related cases are the result of small cell lung cancer (SCLC) [3]. Lymphoma, thymoma, mesothelioma, Ewing’s sarcoma, and a variety of carcinomas including squamous cell carcinoma of the head and neck have all been associated with the development of SIADH [4].

Non-small cell lung cancer (NSCLC) is a very rare cause of SIADH, with only three published cases over the past two decades [5–7]. In one large case series, only 3 of 427 patients with NSCLC (0.7%) were diagnosed with the syndrome [8]. In this report, we present a case of SIADH which developed in a patient with NSCLC following initiation of palliative radiation therapy. This case is unique in that mild hyponatremia worsened concomitantly with clinical improvement and decreased tumour burden, we hypothesize that the release of ADH from malignant cells during tumour lysis, a finding only previously described in SCLC [3].

Serum levels of ADH are elevated in the majority of cases of SIADH, and more severe hyponatremia appears to predict worse prognosis [1]. In these cases, ectopic ADH secretion by malignant cells is the most common mechanism for the development of SIADH. In one study of SCLC, both ADH and atrial natriuretic peptide (ANP) were shown to mediate SIADH, with the quantity of ADH more closely associated with the development of hyponatremia [10]. All SCLC cell lines studied produced ANP and/or ADH mRNA and peptides, while none of 10 NSCLC cell lines produced ADH mRNA or peptide and only two produced ANP mRNA. Selective expression of prepro-AVP-NPII, the precursor of ADH, has also been demonstrated in SCLC, confirming the accepted mechanism of SIADH in this type of malignancy [11].

Non-small cell lung cancers (NSCLC) much more rarely produce polypeptide hormones which are capable of producing paraneoplastic syndrome (PNS). Cases of SIADH associated with NSCLC and other malignancies including squamous cell carcinoma (SCC) have been reported since the 1950s, but the mechanism by which it occurs has not been clarified [12]. Kusuki *et al* suggest that SIADH in the setting of SCC of the head and neck may be caused by the mechanical presence of the tumour itself or treatment via neck dissection [1]. These stimuli can activate carotid and aortic baroreceptors, leading to an increase in ADH secretion [13]. Alternatively, lack of detectable ectopic ADH in SCC may be due to the presence at concentrations below the sensitivity of current assays, storage in an antigenically unrecognizable form, production of a structurally distinct ADH-like hormone, or production of another substance such as neuropeptide Y which can stimulate the posterior pituitary to release ADH or reset the hypothalamic osmostat [1, 13]. In one case, a patient developed SIADH during chemotherapy for SCC of the hypopharynx. It was suspected that cisplatin, over the course of several months and three cycles of therapy, induced an unknown factor which acted on the hypothalamic-pituitary axis to affect the release of ADH [1]. This result is consistent with the literature, which confirms that many chemotherapeutic agents promote ADH release or enhance its action [4].

The time during a patient’s hospital course at which SIADH is diagnosed, therefore, can be highly suggestive of its etiology. Hyponatremia which develops gradually over a long period of time, as described in the previous case, most likely represents stimulation of endogenous ADH secretion rather than production of ectopic hormone. Hyponatremia at presentation is a common feature of malignancy-associated SIADH. In a case series of patients with SCC of the head and neck, the majority of those evaluated before and after surgery had elevated serum ADH levels preoperatively and markedly reduced or normalized levels one week post-operatively [14]. These results suggest ectopic ADH production by tumour as the cause of SIADH.

A recent case report describes a patient with poorly differentiated NSCLC who was severely hyponatremic (115 mmol/l) on presentation [7]. Interestingly, clinical response of this patient’s cancer to MIC chemotherapy correlated with improvement of SIADH-associated hyponatremia. Surgical resection of NSCLC had previously been reported to resolve SIADH [5], this was the first time chemotherapy had been described to successfully treat the conditions in tandem.

The patient in our case was mildly hyponatremic on admission. As above, this finding suggests a low-basal level of ectopic ADH secretion by her mediastinal tumour. After initiation of palliative radiation treatment for NSCLC, her hyponatremia rapidly worsened (from 132 to 123 mmol/l) though she remained asymptomatic. Decreased tumour burden and the resultant symptomatic improvement seemed to be paradoxically linked to declining serum sodium values. Based on the studies previously described, one would expect change in tumour size to be directly proportional to the release of ADH and therefore to the degree of hyponatremia. However, a report of development of hyponatremia following the first course of chemotherapy in a patient with SCLC adds another dimension to the problem [3].

In that case, the patient presented with normal serum electrolytes and renal function. Following VIP chemotherapy, there was a partial radiological response and development of SIADH which resolved after the second cycle. Since hyponatremia did not recur after subsequent courses of the same chemotherapeutic agents, the authors posited that the medications themselves were not responsible for the development of SIADH. Rather, they suggest that the observed hyponatremia was due to ADH release from the tumour during early cell lysis. This phenomenon is very rare, and indeed it is rare in SCLC for a patient to present with normal sodium at diagnosis. However, it is important to understand the possibility of SIADH as both a paraneoplastic and tumour lysis syndrome since the development of hyponatremia during cancer treatment could be incorrectly interpreted as an increase in ectopic ADH production due to ineffective therapy. In actuality, early in the course of treatment, transient hyponatremia can be a sign of highly effective antitumor treatment.

The key to effective management of SIADH is to treat the underlying cause. With infectious etiologies, the appropriate antimicrobials are the treatment of choice, while in medication effect the discontinuation of the offending drug should resolve most cases. In those who have malignant diseases, as with our patient, treatment is antineoplastic therapy. No matter the cause of SIADH, it is always important to evaluate the severity and duration of the hyponatremia, and the presence or absence of symptoms before deciding on the course of treatment.

Patients with severe hyponatremia (sodium less than 120 mmol/L) is related with higher mortality with death rates feat about 50% in elderly patients with sodium levels <115 mmol/l [15].

In symptomatic patients (those with neurological symptoms like seizures or altered mental status thought be secondary to cerebral edema) whose hyponatremia developed over an acute period, defined as 48 h or less, immediate medical treatment is necessary [4, 16]. Although there are no randomized control studies to guide treatment, the approach which has been widely supported recommends raising the patient’s serum sodium level by 1–2 mmol/l by infusing 3% saline [4, 17]. Experts believe that over the first 24 h of treatment, serum sodium levels should not be corrected more than 8–10 mmol/l and over the first 48 h no more than 18–25 mmol/l [4]. The goal of treatment is cessation of neurologic symptoms, after which the correction rate can be reduced.

Asymptomatic patients have a lower risk of neurologic sequela, but can still develop osmotic demyelination syndrome if rapidly corrected [4]. The goal for treatment in these cases is very gradual correction of their hyponatremia. The best approach is to begin with fluid restriction based on the urinary and plasma electrolytes, after which other medications can be tried.

Oral medications that can be tried include oral urea (30 g a day), which increases urinary solute and therefore enhances water secretion [9, 16]. Oral demeclocylcine (300–600 mg twice daily) can also be used. Demecolcycline is a tetracycline derivative that induces diabetes insipidus by reducing the collecting tubules response to ADH [9, 16]. Oral salt tablets can be administered at a dose of 9 g daily in conjunction with furosemide 20 mg twice daily [5, 9]. Furosemide decreases the sodium chloride re-absorption in the thick ascending limb of the loop of Henle, thereby enhancing the effect of the salt tablets [9].

Oral vasopressin-receptor antagonists selective for V2 receptors (tolvaptan, lixivaptan, and satavaptan) have been studied in two randomized control studies [4, 15]. The studies show that these medications are effective at maintaining normal serum sodium levels. The use of these drugs is limited due to their high cost, as well as causing increased thirst, and rapidly correcting the recipient’s hyponatremia.

## Conclusion

The case we have described is the first in which radiation therapy for NSCLC has been associated with clinical improvement and decreased tumour burden, while concomitantly worsening SIADH via tumour lysis.

Although the phenomenon is not well described, case studies have reported patients with NSCLC who have presented with low-serum sodium levels before beginning treatment, suggesting that some NSCLC produce ectopic ADH. There may be an increased risk of developing SIADH once anti-tumour therapy is begun due to elaboration of ADH by malignant cells undergoing mitotic cell death.

This case demonstrates the importance of considering SIADH as a potential PNS and a possible consequence of tumour lysis in patients undergoing anti-tumour therapy for NSCLC.
